# pH rather than nitrification and urease inhibitors determines the community of ammonia oxidizers in a vegetable soil

**DOI:** 10.1186/s13568-017-0426-x

**Published:** 2017-06-21

**Authors:** Ruijiao Xi, Xi-En Long, Sha Huang, Huaiying Yao

**Affiliations:** 1 Key Laboratory of Urban Environment and Health, Institute of Urban Environment, Chinese Academy of Sciences, Xiamen, 361021 People’s Republic of China; 2 Ningbo Key Laboratory of Urban Environmental Processes and Pollution Control, Ningbo Urban Environment Observation and Research Station—NUEORS, Chinese Academy of Sciences, Ningbo, 315800 People’s Republic of China; 3 University of Chinese Academy of Sciences, Beijing, 100049 People’s Republic of China; 4 Key Laboratory for Green Chemical of Ministry of Education, Wuhan Institute of Technology, Wuhan, 430073 People’s Republic of China

**Keywords:** Soil pH, Ammonia oxidizers, Vegetable soil, Nitrification inhibitors, Urease inhibitors

## Abstract

Nitrification inhibitors and urease inhibitors, such as nitrapyrin and *N*-(*n*-butyl) thiophosphoric triamide (NBPT), can improve the efficiencies of nitrogen fertilizers in cropland. However, their effects on ammonia-oxidizing archaea (AOA) and ammonia-oxidizing bacteria (AOB) across different soil pH levels are still unclear. In the present work, vegetable soils at four pH levels were tested to determine the impacts of nitrification and urease inhibitors on the nitrification activities, abundances and diversities of ammonia oxidizers at different pHs by real-time PCR, terminal restriction fragment length polymorphism (T-RFLP) and clone sequence analysis. The analyses of the abundance of ammonia oxidizers and net nitrification rate suggested that AOA was the dominate ammonia oxidizer and the key driver of nitrification in acidic soil. The relationships between pH and ammonia oxidizer abundance indicated that soil pH dominantly controlled the abundance of AOA but not that of AOB. The T-RFLP results suggested that soil pH could significantly affect the AOA and AOB community structure. Nitrapyrin decreased the net nitrification rate and inhibited the abundance of bacterial *amoA* genes in this vegetable soil, but exhibited no effect on that of the archaeal *amoA* genes. In contrast, NBPT just lagged the hydrolysis of urea and kept low NH_4_^+^-N levels in the soil at the early stage. It exhibited no or slight effects on the abundance and community structure of ammonia oxidizers. These results indicated that soil pH, rather than the application of urea, nitrapyrin and NBPT, was a critical factor influencing the abundance and community structure of AOA and AOB.

**Electronic supplementary material:**

The online version of this article (doi:10.1186/s13568-017-0426-x) contains supplementary material, which is available to authorized users.

## Introduction

Nitrification is a necessary transition to convert ammonia to nitrate in soils, which can significantly affect the ecological system. Nitrogen losses by the leaching of nitrate and the emissions of N_2_O have become more and more severe in last several years (IPCC 2007). Although it occurs in the terrestrial environments at very low concentrations, the concentration of nitrous oxide has grown 18.5% since the preindustrial period (IPCC 2007). Among total global nitrous oxide emitted by various sources and human activities, soils account for about 62% (Thomson et al. 2012). In addition, the nitrate unabsorbed by plants in soils can be leached due to its mobility in soil. To reduce nitrogen loss and increase the nitrogen fertilizer use efficiency, nitrification inhibitors (NIs) and urease inhibitors (UIs) are usually applied to agricultural systems (Cui et al. 2013; Sanz-Cobena et al. 2011).

Among the nitrification inhibitors, nitrapyrin or N-serve (2-chloro-6-(trichloromethyl) pyridine) is an individual inhibitor of ammonium oxidation (Hughes and Welch 1970). It inhibits the first step of nitrification from ammonia to nitrite (Kangatharalingam and Priscu 1993) by targeting ammonia monooxygenase (AMO) that catalyzes the conversion of NH_3_ to NH_2_OH (Arp et al. 2002). *N*-(*n*-butyl) thiophosphoric triamide (NBPT) is one of the most effective urease inhibitor (Bremner and Chai 1986; Bronson et al. 1989). It can lower the hydrolysis rate and volatilization loss of urea as it is applied to soils at high concentrations (Antisari et al. 1996; Watson et al. 1994). NBPT is transformed into *N*-(butyl) phosphoric triamide (NBPTO) as it is directly applied to soil (Creason et al. 1990). Both NBPT and NBPTO inhibit urease activity by competing with urea molecules for the enzyme Ni receptor sites (Kolodziej 1994).

In general, ammonia-oxidizing *Archaea* (AOA) and ammonia-oxidizing *Bacteria* (AOB) are the key drivers of the ammonia oxidation in soil (Jin et al. 2010; Li and Gu 2013). AOA and AOB use the same substrate for energy metabolism, but differ in their biochemistries and physiologic properties, such as the molecular and cellular features (Lehtovirta-Morley et al. 2011; Kim et al. 2012). The differences of AOA and AOB membrane structures lead to different membrane permeability, and thus cause different nitrification activities (Shen et al. 2008; Schouten et al. 2000). In addition, they occupy different ecological niches due to their dissimilar sensitivity to soil properties, such as nitrogen concentration, pH, water content, and so on (Morimoto et al. 2011; Shen et al. 2008). By comparing the inhibitory effects of allylthiourea (ATU) and nitrapyrin on ammonia oxidizers, Jäntti et al. (2013) concluded that ATU was not a good nitrification inhibitor for the communities containing AOA and nitrapyrin exhibited good inhibitory effects in presences of both AOA and AOB. Lehtovirta-Morley et al. (2013) investigated the inhibitory effects of nitrapyrin at different concentrations on the growth of ammonia oxidizers in soil and liquid cultures at pH 4.5 and found that the abundance of *amoA* varied with the nitrapyrin concentration and culture environment. (Belser and Schmidt 1981) reported the inhibitory effects of nitrapyrin on seven strains of ammonia oxidizers. Although nitrapyrin has been well-studied for years, its effects on the diversity and richness of ammonia oxidizers across different soil pH levels have never been reported. In previous study real-time PCR was used regularly based on *amoA* gene copies, but there has been very little concern on the community shifts of ammonia oxidizers in the presence of NIs and UIs (Liu et al. 2015). For example, Shen et al. (2013) found that the inhibitory effect of nitrapyrin on *Ca. Nitrososphaera* was more effective than that on *N. multiformis* by cultivating two representative strains of AOA and AOB and calculating the effective concentration 50 (EC_50_). It was shown that nitrapyrin could increase the ammonium retention and decrease the gross nitrification at 40 °C, but had no effect on the abundances of the bacterial ammonia oxidizer genes (Fisk et al. 2015). Other studies on the effect of nitrapyrin on *amoA* gene copies, nitrous oxide emissions also have been reported (Regina et al. 1998).

Ammonia-oxidizing microorganisms are influenced by many environmental factors, like substrate concentration, land utilization, organic matter, temperature, pH, oxygen concentration, and so on (Di et al. 2009; Ying et al. 2010; Abell et al. 2011), among which the soil pH has a particularly important effect on the abundance and diversity of ammonia oxidizers (Liu et al. 2015; Nicol et al. 2008). An examination of 65 soil samples collected from different regions and ecosystems indicated that pH drove the distribution of ammonia oxidizers and the AOA/AOB ratio declined with the increase of soil pH (Hu et al. 2013). AOA exhibited a more competitive advantage than AOB in acidic soils. In addition, the diversity of AOA was mainly affected by pH at pHs below 3.5 and not significantly influenced by the soil type and land-use method (Stempfhuber et al. 2015). Nicol et al. (2008) confirmed that soil pH determined the phylotype distribution of bacterial and archaeal ammonia oxidizers. Li et al. (2015) also reported that the ammonia oxidizers community structure and nitrification activity were significantly affected by soil pH. The aim of our work was to investigate the short-term effects of nitrapyrin and NBPT on nitrification and the abundance and community structure of AOA and AOB in a vegetable soil across a pH-gradient. Four treatments at four pH levels in the range of 3.97–7.04 were conducted. Molecular biological technologies including quantitative PCR, terminal restriction fragment length polymorphism (T-RFLP) and clone libraries were used in our study.

## Materials and methods

### Sample collection and microcosm incubation

Soil samples (0–20 cm depth) were collected from a vegetable field in Ningbo (121°51′N, 29°54′E), Zhejiang Province in eastern China. The sampling site was planted with Chinese cabbages (*Brassica campestris* spp. *Pekinensis*) for over 10 years and fertilized with average 450 kg N ha^−1^ year^−1^. The soil was classified as red soil (equivalent to Ultisols in US soil taxonomy), and developed on quaternary red earth. Mean annual rainfall in this area is 1300–1500 mm and mean temperature is 16.6 °C. The vegetable field is a sandy loam soil composed of 13.97% clay, 21.75% silt and 64.28% sand with a pH of 3.97, total nitrogen (TN) content of 0.64%, microbial biomass carbon (MBC) of 382.12 mg kg^−1^, microbial biomass nitrogen (MBN) contents of 63.04 mg kg^−1^, and potential nitrification rate (PNR) of 0.44 mg NO_3_^−^-N kg^−1^ h^−1^. The collected soil samples were ground to pass through a 2-mm sieve after air-dried. The water-holding capacity of the soil was determined to assure the consistent water content.

The pH of the soil sample was adjusted to 3.97, 4.82, 6.07 and 7.04 by CaCO_3_, separately. Four treatments including control, 200 mg kg^−1^ urea-N, 200 mg kg^−1^ urea-N + nitrapyrin (0.1% of urea-N) and 200 mg kg^−1^ urea-N + NBPT (2% of urea-N) were applied to the soil of each pH level in triplicates. Soil samples were thoroughly mixed with the composite of urea, nitrapyrin and NBPT, and put in a plastic jar, then kept the 50% water-holding capacity (WHC). The plastic jar was covered with a plastic lid with aeration holes to keep an aerobic environment. The soil samples were then incubated in the dark at 25 °C for 28 days in a chamber. The soil moisture was maintained at 50% WHC by weighing the soil sample once a day.

After the fertilizer, nitrapyrin and NBPT were supplied, the destructive sampling of ~20 g soil was conducted in triplicates at day 1, 3, 7, 14, 21 and 28. About 5.0 g of soil samples were stored at −80 °C for DNA extraction, the rest of the fresh soil samples was used for further chemical analysis.

### Soil chemical analysis

The soil NO_3_^−^-N and NH_4_^+^-N were extracted from fresh soil samples using 2 mol l^−1^ KCl (soil/KCl, 1:5) and measured with a flow injection analyzer (FLA star 5000 Analyzer, Foss, Denmark). MBC and MBN were determined by the fumigation-extraction method (Wu et al. 1990). Briefly, the soil samples were fumigated with CHCl_3_ for 24 h at room temperature in the dark. The fumigated samples and samples without fumigated were extracted with 0.5 M K_2_SO_4_ for 30 min on a shaker and filtrated. The filtrates were measured on an automated TOC Analyzer (TOC-500, Japan). PNR was measured in triplicates by the shaken-slurry method (Yao et al. 2011). Fifteen grams of fresh soil samples were mixed with 7.5 ml of 0.2 M KH_2_PO_4_, 17.5 ml of 0.2 M K_2_HPO_4_ and 75 ml of 0.05 M (NH_4_)_2_SO_4_, respectively, and incubated in the dark at 25 °C for 24 h on a 180 rpm shaker. Suspension aliquots of 10 ml were sampled at 2, 4, 22 and 24 h incubation, respectively, and immediately analyzed on the continuous flow analyzer to determine their nitrate concentrations. The measurements of other soil properties were the same as described by Zhang et al. (2012).

The net nitrification rate (*n*) was calculated by the formula presented by Persson and Wirén (1995) as follows:

$$n\left( {{\text{mg N kg}}^{ - 1} {\text{soil day}}^{ - 1} } \right) = \frac{{({\text{NO}}_{3}^{ - } - {\text{N}})_{t2} - \left( {{\text{NO}}_{3}^{ - } - {\text{N}}} \right)_{t1} }}{t}$$

where (NO_3_^−^-N)_*t*2_ and (NO_3_^−^-N)_*t*1_ are the concentrations of NO_3_^−^-N in the soil at time *t*2 and time *t*1 respectively, and *t* is the number of days between *t*2 and *t*1.

### DNA extraction

DNA was extracted from 500 mg frozen soil using the FastDNA^®^ SPIN Kit for Soil (Bio 101, Vista, CA) according to the manufacturer’s instruction, immediately diluted ten times and stored at −20 °C for molecular analyses. DNA concentration was measured on a NanoDrop ND-1000 UV–vis spectrophotometer (NanoDrop^®^, USA).

### Real-time PCR assay of *amoA* genes

Quantitative PCR of *amoA* genes was conducted on a Light Cycler 480 real-time PCR detection system (Roche480, USA). Standard plasmids of AOA and AOB were constructed and diluted one- to nine-folds to construct the standard curve. Two different pairs of primers were used to target the AOA and AOB respectively (Additional file 1: Table S1). Each PCR reaction was performed in a 20-μl reaction mixtures consisting of 0.5 μM of each primer, 10 μl of SYBR^®^ Premix, 1 μl of tenfold dilution DNA template, 0.5 μl of bovine serum albumin (BSA, 20 mg·ml^−1^), and the residual volume replenished by deionized water. For quantification of AOA and AOB, the amplification efficiencies were in the range from 90 to 96% and the correlation coefficient (r^2^) of the determination ranged from 0.95 to 0.99 for all of the standard curves.

### T-RFLP of *amoA* genes for ammonia oxidizers

For analysis of the ammonia oxidizers community, T-RFLP was conducted from the soils of all treatments at day 28. Primers used in the qPCR with the forward primer marked with 6-carboxyfluorescein (FAM) (Additional file 1: Table S1) were used for the T-RFLP (Ying et al. 2010). The AOA and AOB samples were digested with restriction with HpyCH4V and MspI, respectively. The PCR products were purified using the concrete method presented by Yao et al. (2011). Fragments with sizes longer than 50 bp and percentages higher than 1% were kept for cluster analysis and the rest fragments were eliminated.

### Cloning and sequencing

To identify the T-RFs, the AOA and AOB clone libraries from all the soil samples at day 28 were constructed with same primers CrenamoA23f/616r and amoA-1F/2R used in the qPCR analysis, but the different enzyme. One hundred clones were selected from these two clone libraries. The sequences displaying less than 2% nucleotide dissimilarities with each other were grouped into the different operational taxonomic unit (OTU). Representative sequences selected from each OTU were then used to build phylogenetic trees. Phylogenetic trees were constructed with Mega software (Tamura et al. 2013). The sequences of AOA and AOB were grouped into 6 and 16 OTUs, respectively. Eight representative sequences of AOA and 23 representative sequences of AOB were selected. Sequences that were analogous to the representative sequences most were selected from the GenBank to construct the phylogenetic tree.

### Statistical analysis

To compare the *amoA* genes abundance of AOA and AOB among all treatments, data were analyzed using ANOVA with SPSS 19.0 software (IBM, USA). Pearson correlation analysis (*P* < 0.05) was also performed in SPSS 19.0 software. In order to anticipate the variations among the ammonia oxidizer community structures of different treatments and pHs, the T-RFLP data were analyzed in the CANOCO version 4.5. The construction of phylogenetic trees used MEGA version 6.0 software (Tamura et al. 2013). A 500 replicates bootstrap analysis was performed to evaluate the cluster stability.

### Accession numbers of nucleotide sequences

The nucleotide sequences accession numbers of AOA and AOB were KX683109–KX683208 and KY073755–KY073854, respectively.

## Results

### Concentrations of inorganic nitrogen and net nitrification rate

**Fig. 1:**
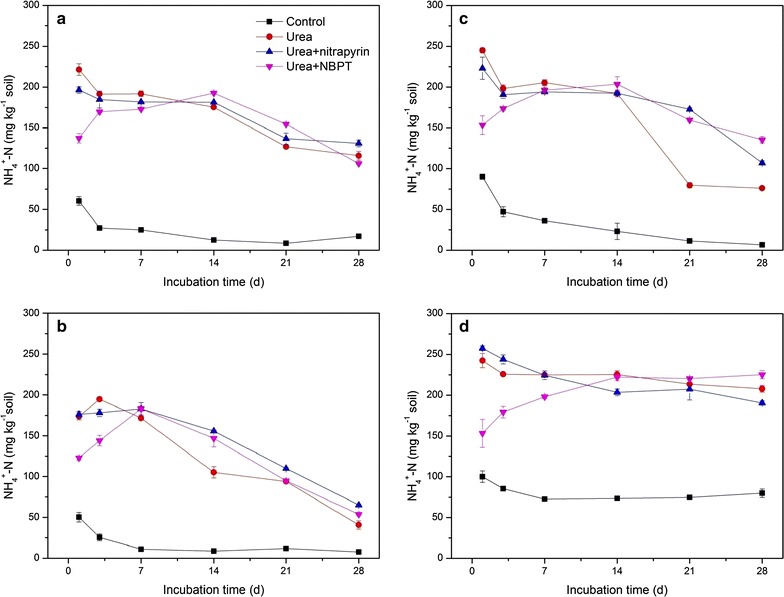
The variation of NH_4_^+^-N concentrations as affected by applications of urea, nitrification inhibitor (nitrapyrin) and urease inhibitor (NBPT) at different pH levels. *Error bars* indicate standard deviation of mean (*n* = 3). **a** pH 3.97; **b** pH 4.82; **c** pH 6.07; **d** pH 7.04

**Fig. 2:**
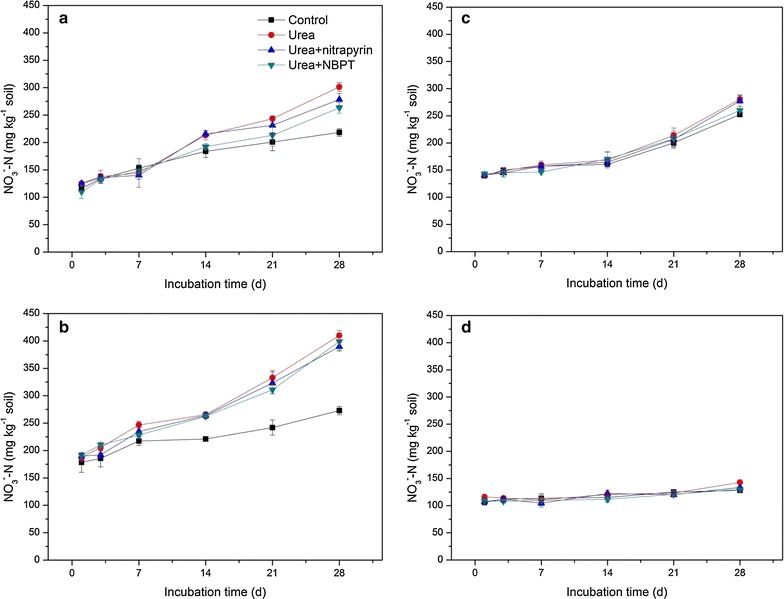
The variation of NO_3_^−^-N concentrations as affected by applications of urea, nitrification inhibitor (nitrapyrin) and urease inhibitor (NBPT) at different pH levels. *Error bars* indicate standard deviation of mean (*n* = 3). **a** pH 3.97; **b** pH 4.82; **c** pH 6.07; **d** pH 7.04

The temporal changes of NH_4_^+^-N concentrations at four pH levels exhibited a similar trend. Compared with control soil, the soil treated with urea only contained a significantly higher NH_4_^+^-N concentration (Fig. 1). The NH_4_^+^ concentration in urea treatment rapidly decreased after day 3 at all pH levels except for pH 7.04 and then remained higher than that in the control soil. Inhibitors exhibited different inhibiting effects on the soils of different pH levels. The NH_4_^+^-N concentrations in urea + NBPT treatment were lower than in urea treatment at the early stage (Fig. 1). The NO_3_^−^-N concentrations in soils of four pH levels gradually increased from day 1 to day 28. Control soil contained the lowest concentration of NO_3_^−^-N and the highest concentration was found in soil of urea treatment. The NO_3_^−^-N concentrations in the soils of four treatments remained relatively stable at pH 7.04 during the 28-day incubation (Fig. 2). The NO_3_^−^-N concentrations in urea + nitrapyrin treatment at the four pH levels were ~8.2, ~5.2, ~1.1 and ~6.9%, respectively, lower than those in the soils of urea treatment at corresponding pH levels at day 28 (Fig. 2). The NO_3_^−^-N concentrations in the soils of urea + NBPT treatment at the four pH levels were about ~14.5, ~2.7, ~7.9 and ~9.7%, also lower than those in soils of urea treatment at corresponding pH levels at day 28. Both inhibitors had a significant effect on nitrate concentration at pH 3.97 (Fig. 2).

Control soil exhibited the lowest net nitrification rates at all testing pHs. The application of urea increased the net nitrification rates 72.3, 134.8, 24.4, and 23.8%, respectively (Table 1). Net nitrification rates in urea + nitrapyrin and urea + NBPT treatments were higher than that in the control soil and lower than that in the soil of urea treatment at all testing pH levels except for pH 7.04. Net nitrification rate at pH 7.04 was the lowest for the soils of all treatments (Table 1). The correlation analysis suggested that net nitrification rate and soil pH in the tested soil existed a significant relationship (r = −0.736, *P* < 0.01) (Table 2).

**Table 1:** Net nitrification rate (mean ± SE) of each treatment during incubation time

pH	Net nitrification rate (mg kg^−1^ day^−1^)			
	Control	Urea	Urea + nitrapyrin	Urea + NBPT
3.97	3.79 ± 0.11c	6.56 ± 0.13a	5.58 ± 0.09b	5.65 ± 0.07b
4.82	3.55 ± 0.09d	8.21 ± 0.09a	7.34 ± 0.08c	7.68 ± 0.10b
6.07	4.18 ± 0.05d	5.21 ± 0.10a	5.02 ± 0.10b	4.33 ± 0.09c
7.04	0.80 ± 0.09b	0.99 ± 0.07a	0.97 ± 0.01a	0.79 ± 0.06b

Values are mean or mean ± standard deviation (*n* = 3). Values within the same row followed by the different letters indicate significant difference (*P* < 0.05)

**Table 2:** Pearson correlation between target gene copies, pH and net nitrification rate

Item	AOA	AOB	pH	Net nitrification rate
AOA	1	−0.138	−0.926**	0.790**
AOB	−0.138	1	0.192	−0.282
pH	−0.926**	0.192	1	−0.736**
Net nitrification rate	0.790**	−0.282	−0.736**	1

** Correlation is significant at the 0.01 level (two-tailed)

### *amoA* gene abundances

**Fig. 3:**
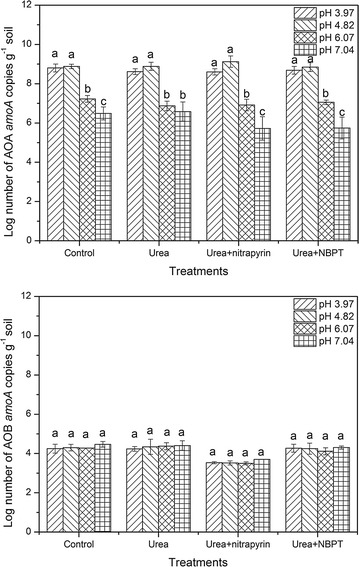
Log number of AOA and AOB *amoA* copies in four different treatments (control; urea; urea + nitrapyrin; urea + NBPT) at different pH levels. *Error bars* indicate standard errors of three replicates, *different small letters* indicate the significant difference within the same treatment at different pH levels (*P* < 0.05)

The variation in the AOA and AOB population sizes in the soils of different treatments was determined on day 28 (Fig. 3; Additional file 1: Figure S1). AOA *amoA* gene copy numbers were in the range from 9.49 × 10^4^ to 3.43 × 10^9^ g^−1^ dry soil. The highest AOA population (1.01 × 10^9^ g^−1^dry soil) was found at soil pH 4.82 and the lowest (3.02 × 10^6^ g^−1^dry soil) was observed at soil pH 7.04. No differences were found among the AOA *amoA* gene copy numbers of four treatments at the same pH level. The AOA abundance exhibited a positive correlation with the net nitrification rate (r = 0.790, *P* < 0.01) and a negative correlation with pH (r = −0.926, *P* < 0.01) (Table 2). The AOB *amoA* gene copy numbers ranged from 9.80 × 10^3^ to 2.93 × 10^4^ g^−1^ dry soil. The quantitative PCR analysis indicated that the AOA abundance was much higher than that of AOB abundance in the corresponding soil. No significant differences among the AOB abundances in the soils of all treatments except for the nitrapyrin treatment were observed (Fig. 3; Additional file 1: Figure S1).

### AOA and AOB communities

**Fig. 4:**
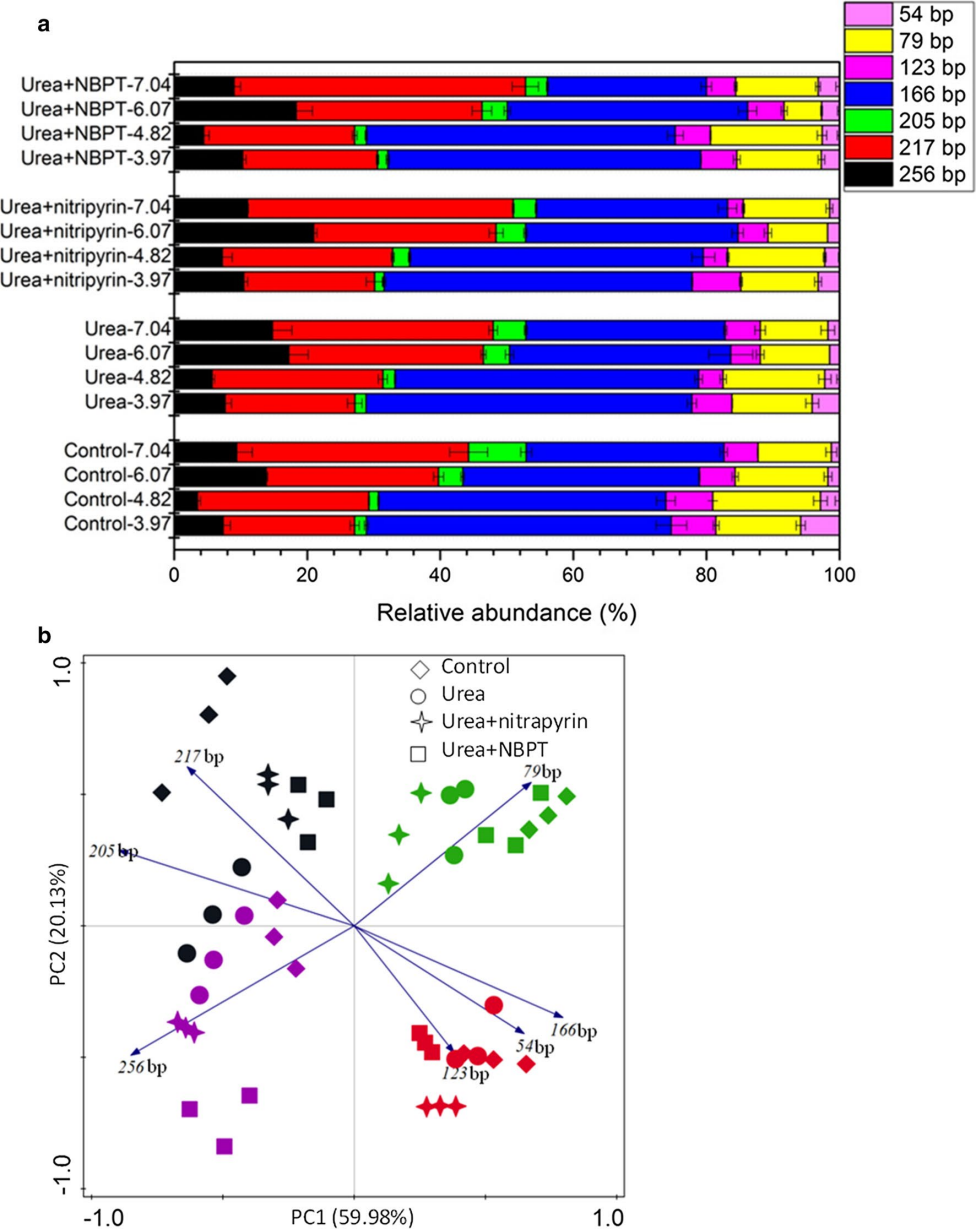
Abundance (**a**) and principle component analysis (**b**) of AOA T-RFs in vegetable soils treated with control, urea, urea + nitrapyrin, urea + NBPT at different pH. *Error bars* indicate standard errors of three replicates. Different pH represented by different colors, *red*, *green*, *purple* and *black color* indicate treatments at pH 3.97, pH 4.82, pH 6.07 and pH 7.04, respectively

**Fig. 5:**
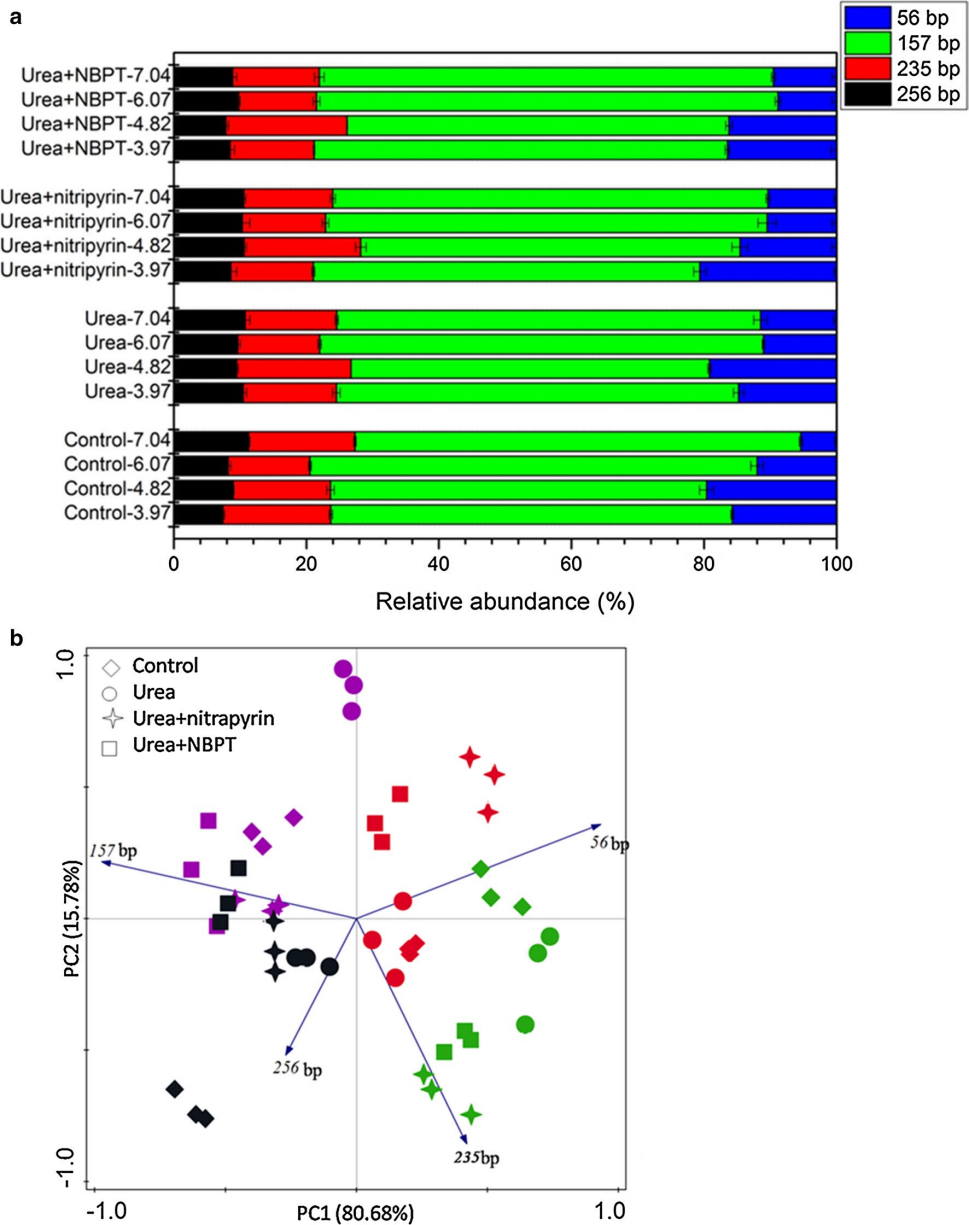
Abundance (**a**) and principle component analysis (**b**) of AOB T-RFs in vegetable soils treated with control, urea, urea + nitrapyrin, urea + NBPT at different pH. *Error bars* indicate standard errors of three replicates. Different pH represented by different colors, *red*, *green*, *purple* and *black color* indicate treatments at pH 3.97, pH 4.82, pH 6.07 and pH 7.04, respectively

The AOA and AOB communities in the vegetable soils of different treatments and at different pH levels were determined by T-RFLP (Figs. 4, 5). Seven AOA T-RFs were obtained, among which T-RF 166 bp (23.90–48.97%) and T-RF 217 bp (19.49–43.80%) accounted for the most proportion of the community. The relative abundance of AOA T-RF 166 bp was negatively correlated with soil pH (r = −0.963, *P* < 0.01) and the relative abundances of AOA T-RFs 217 bp and 205 bp exhibited extreme and positive correlations with soil pH (r = 0.905, *P* < 0.01; r = 0.778, *P* < 0.01, respectively) (Additional file 1: Table S2). PCA analyses further confirmed these community shifts among all treatments (Fig. 4b), and showed that two PCA axes explained 80.11% of the observed structure. A significant separation was found between treatments at pH 3.97 and 4.82 and those at pH 6.07 and 7.04 along PC1, and a distinct separation between treatments at pH 3.97 and those at pH 4.82 was observed along PC2. However, the applications of urea, nitrapyrin and NBPT exhibited no significant effect on the AOA T-RFLP pattern.

Four major AOB T-RFs of 56, 157, 235, and 256 bp were obtained. T-RF 157 bp accounted for about 62.73% of total T-RFs and its abundance had a significant and positive relationship with soil pH (r = 0.721, *P* < 0.01). The abundance of AOB T-RF 56 bp was negatively correlated to soil pH (r = −0.825, *P* < 0.01) (Additional file 1: Table S3). The two axes of PCA can explain 96.46% of the observed structures (Fig. 5b). The changes in the relative abundances of T-RFs 157 and 56 bp were the dominated alteration. The samples at pH 3.97 and 4.82 were separated from the samples at pH 6.07 and 7.04 along PC1 at a significant level. Similar to AOA, no significant separation was found between the four treatments at the same pH.

### Genotypes of ammonia oxidizers and their phylogenetic tree analysis

**Fig. 6:**
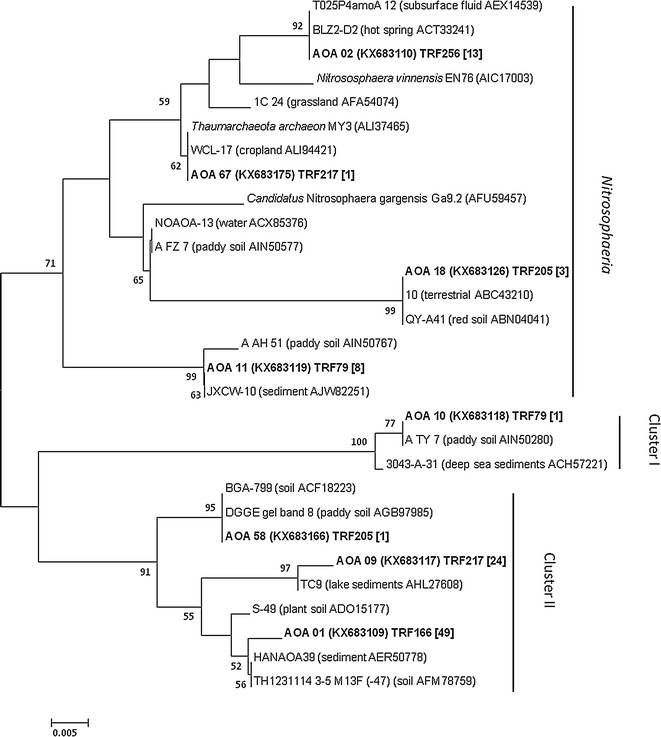
Neighbor-joining phylogenetic tree of AOA *amoA* gene sequences retrieved from the vegetable soil. Sequences from this study are shown in *bold* and are described as clone name (accession number) T-RF size. Bootstrap values (>50%) are indicated at *branch points*. Reference sequences are described as clone name (environment, accession number). The *number in bracket* means clones. The *scale bar* represents 0.5% estimated sequence divergence

**Fig. 7:**
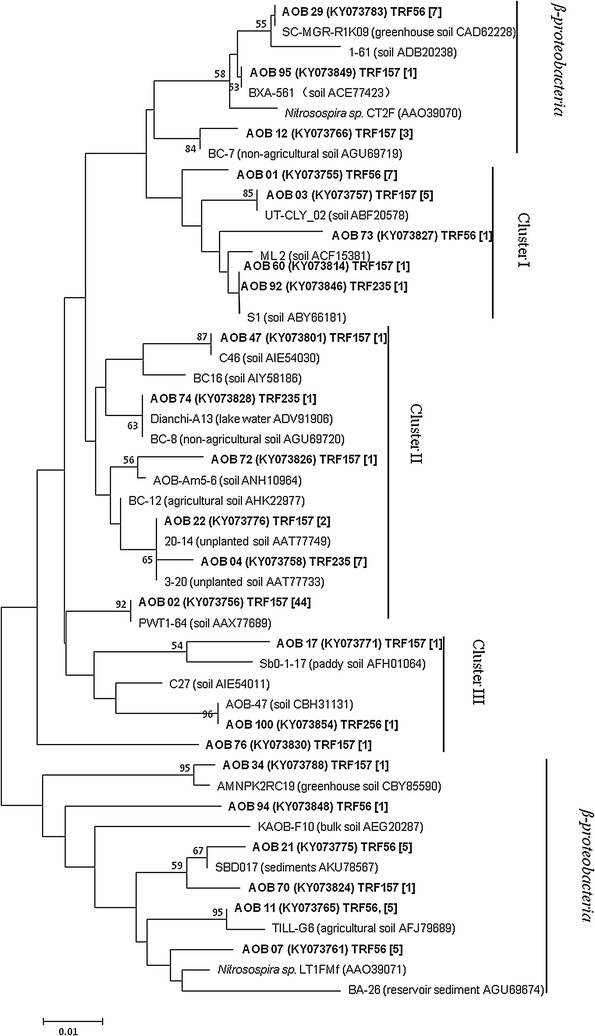
Neighbor-joining phylogenetic tree of AOB *amoA* gene sequences retrieved from the vegetable soil. Sequences from this study are shown in *bold* and are described as clone name (accession number) T-RF size. Bootstrap values (>50%) are indicated at *branch points*. Reference sequences are described as clone name (environment, accession number). The *number in bracket* means clones. The *scale bar* represents 1% estimated sequence divergence

The trees of *amoA* amino acid sequences and related GenBank sequences are shown in Figs. 6 and 7, respectively. There were 6 OTUs and 16 OTUs identified for AOA and AOB, respectively (Additional file 1: Table S4). Two representative OTUs (OTU 01 and OTU02) (Additional file 1: Table S4) and 8 representative sequences of AOA were included into three different clusters. Half of the representative sequences belonged to group *Nitrososphaera* and the rest were grouped into cluster I and cluster II (Fig. 6). The representative T-RFs 217 and 166 bp were related to OTU02 (KX683117) and OTU01 (KX683109) (Additional file 1: Table S4), respectively. One representative OTU (OTU05) of bacterial *amoA* genes was selected (Additional file 1: Table S4) and 23 AOB representative sequences were chosen and classified into five different clusters. Two of the clusters belonged to *β*-*proteobacteria* and the rest belonged to cluster I–III. Nine of the representative sequences belonged to *β*-*proteobacteria* and the rest 14 representative sequences were grouped into cluster I–III (Fig. 7). The representative T-RF 157 bp was most related to OTU05 (KY073756) (Additional file 1: Table S4).

The results of T-RFLP analyses and phylogenetic trees of AOA indicated that the dominant T-RFs 166 and 217 bp belonged to cluster II were the predominant AOA genotypes in the soils of all treatments (Additional file 1: Table S4, Fig. 6). T-RF 205 bp fell into both *Nitrososphaera* and Cluster II, but mainly into *Nitrososphaera*. The phylogenetic tree of AOB indicated that all *amoA* gene sequences of bacteria in this vegetable soil belonged into *β*-*proteobacteria* and cluster I–III (Fig. 7). T-RF 157 bp spread the whole clone library, but mostly belonged to cluster II. T-RF 56 bp fell into cluster I and *β*-*proteobacteria.*

## Discussion

### Abundances and activities of ammonia oxidizers

In the vegetable soil tested in the present work, the abundance of AOA was significantly higher than that of AOB, in agreement with previous findings (Chen et al. 2011, 2015; Gubry-Rangin et al. 2010). In addition, the abundance of AOA was negatively correlated to soil pH, indicating the preference of AOA to acidic soil. The AOA abundance sharply decreased at pH 7.04, indicating that AOA was inactivated in neutral soil. It might be explained by the competition between AOA and AOB for the limited energy source, especially the limited ammonia in the acidic soil (He et al. 2012). Zhang et al. (2012) found the important role of AOA in acidic soils was attributed to the low-pH-reduced availability of ammonia and high substrate affinity of AOA. In addition, the positive correlation between AOA abundance and net nitrification rate indicated that the ammonia oxidation was mainly attributed to AOA (Table 2) and AOA was the main driver of nitrification in the vegetable soil examined. The abundances of AOB in the soils of same fertilizer treatment at different pH levels showed no significant differences, indicating that the abundance of AOB was less affected by pH. The results were mainly due to the high AOA to AOB ratio in the original soil (Fig. 3).

### AOA and AOB communities

Soil pH is a very important factor influencing the distribution of ammonia oxidizers. In the present work, the T-RFLP analysis suggested that the AOA and AOB community structure varied and were correlated with soil pH (Figs. 4, 5). It has been reported that soil pH exhibits similar effects on the AOA and AOB community structure in Chinese tea orchard soils (Yao et al. 2011). Nicol et al. (2008) also revealed that the community structure changed across a soil pH gradient with specific species in acidic and neutral soils. Studies have shown that the nitrogen fertilizer and nitrification inhibitors can affect the community composition of ammonia oxidizers (Yao et al. 2011, 2016; Mahmood and Prosser 2006). However, the T-RFLP and PCA analyses showed that urea, nitrapyrin and NBPT had less effects on the community composition of ammonia oxidizers than soil pH (Figs. 4, 5). Therefore, it can be concluded that the community structure of AOA and AOB are more impressionable to soil pH than to urea, nitrapyrin and NBPT. Shifts in the ammonia oxidizer community structure might be due to the variation of soil pH. The relative abundances of AOA T-RFs 217, 205 and 166 bp and AOB T-RFs 157 and 56 bp were related to soil pH. AOA T-RFs 217 and 205 bp and AOB T-RF 157 bp were more suitable to acidic soil and AOA T-RF 166 bp and AOB T-RF 56 bp were more suitable to neutral soil. This is similar to the findings in Chinese tea orchard soils where some T-RFs are correlated to soil pH and the relative abundances of AOA T-RF 166 bp and AOB T-RF 156 bp decrease and those of AOA T-RFs 205 bp and 217 bp increase with the increase of soil pH. It has also been reported in the National Soil Inventory of Scotland that AOA T-RF 217 bp is relevant to high pH (Yao et al. 2013). Our results are consistent with these findings that soil pH is the major driver of AOA and AOB community structure.

### Effects of nitrapyrin on soil inorganic nitrogen availability and abundance of ammonia oxidizers

Nitrapyrin is the most well-studied highly effective nitrification inhibitor. It can keep nitrogen in the form of ammonia by chelating copper components of the cytochrome oxidase involved in ammonia oxidation (Subbarao et al. 2006). The concentrations of NO_3_^−^-N in the soils of urea + nitrapyrin treatment across the pH gradient at day 28 were lower than those in the soils of urea treatment at corresponding pH levels, and the net nitrification rates in the soils of urea + nitrapyrin treatment across the pH gradient were also lower than those in the soils of urea treatment (Table 1), indicating that nitrapyrin inhibited nitrification and its inhibitory effect varied with soil pH. This is consistent with the finding reported previously (Hall 1984; Chancy and Kamprath 1982; Degenhardt et al. 2016; Sims and MacKown 1987; Touchton et al. 1979; Hendrickson and Keeney 1979). For example, (Hendrickson and Keeney 1979) found that the inhibitory efficiency of nitrapyrin increased with soil pH. However, the affecting mechanism of pH on the inhibitory efficiency of nitrapyrin is not clear. For the abundance of *amoA* genes, the abundance of AOB *amoA* genes decreased significantly in urea + nitrapyrin treatment in comparison with treatments without nitrapyrin (Additional file 1: Figure S1), indicating that nitrapyrin inhibited the growth of AOB. However, no significant effect of nitrapyrin on AOA abundance was observed in the vegetable soil. The inhibition of the abundance of ammonia oxidizers by nitrapyrin have been rarely studied. Cui et al. (2013) demonstrated that nitrapyrin reduced the AOB abundances in alluvial soil and paddy soil. Fisk et al. (2015) indicated that the abundance of bacterial *amoA* genes could be decreased by nitrapyrin at 20 °C. Our results further confirmed that nitrapyrin could significantly decrease the abundance of AOB, but not that of AOA. This can be justified by the different cell membrane compositions of AOA and AOB, which affects the permeability of membranes, and thus differ in their sensitivity to the NIs (Ruser and Schulz 2015).

### Effects of NBPT on soil inorganic nitrogen availability and ammonia oxidizers

NBPT as a urease inhibitor can effectively delay the hydrolysis of urea (McCarty et al. 1989; Wang et al. 1991; Kawakami et al. 2012). Our results indicated that the addition of NBPT reduced the NO_3_^−^-N concentration and net nitrification rate in the tested soil. However, the decreasing degree of the net nitrification rate varied with soil pH (Table 1), indicating that NBPT could inhibit the hydrolysis of urea and the inhibition efficiency was affected by soil pH. Hendrickson and Douglass (1993) revealed that pH was a key factor for NBPT to control the urea hydrolysis and both NBPT and BNPO (an oxon analog of NBPT) could inhibit the hydrolysis of urea more effectively in neutral soils than in acidic soils. However, Beyrouty et al. (1988) found that the effect of NBPT was rarely influenced by soil pH and NBPT could be applied to both acidic and neutral soils. Our results indicated that NBPT was a much more effective urease inhibitor at pH 3.97 than at other pHs. Therefore, the inhibition efficiency of NBPT might be also influenced by other environmental factors. Further study is essential to definite the transformation of NBPT under different environmental conditions. NBPT exhibited no significant effects on the abundance and community structure of ammonia oxidizers. The effects of NBPT on bacteria, fungi and actinomycetes have been well studied. For example, Zhao et al. (2007) found that high concentrations of NBPT could inhibit the growths of bacteria and actinomycete. Song and Sun (2006) reported that NBPT could promote the growth of soil bacteria, actinomycetes and fungi. However, NBPT has shown no effects on growth of ammonia oxidizers, consistent with our results.

In conclusion, our results indicated that nitrapyrin could inhibit the growth of AOB but not AOA. AOA were affected by pH more significantly than AOB. AOA was the dominate ammonia oxidizers and drove the nitrification in acidic soils. NBPT was able to inhibit the urea hydrolysis, but exhibited no significant effect on the abundance and community structure of ammonia oxidizers. Community populations of AOA and AOB were more susceptive to pH than to NIs and UIs. Future work will be focused on the roles of AOA and AOB in autotrophic nitrifying activity using DNA-SIP technologies.

## Additional file

**Additional file 1: Table S1.** Primers of AOA and AOB used for molecular analyses. **Table S2.** Pearson correlation between pH and the relative abundance of archaeal ammonia oxidizer TRFs. **Table S3.** Pearson correlation between pH and the relative abundance of bacterial ammonia oxidizer TRFs. **Table S4.** Genotype patterns based on the clone libraries of *amoA* genes. **Figure S1.** Log number of AOA and AOB *amoA* copies in four different treatments (control; urea; urea+nitrapyrin; urea+NBPT) at different pH levels. *Error bars* indicate standard errors of three replicates, *different capital letters* indicate the significant difference within different treatments at same pH level (*P* < 0.05).

## Abbreviations

NBPT: *N*-(*n*-butyl) thiophosphoric triamide
AOA: ammonia-oxidizing archaea
AOB: ammonia-oxidizing bacteria
T-RFLP: terminal restriction fragment length polymorphism
N_2_O: nitrous oxide
Nis: nitrification inhibitors
UIs: urease inhibitors
N-serve: 2-chloro-6-(trichloromethyl) pyridine
AMO: ammonia monooxygenase
NBPTO: *N*-(butyl) phosphoric triamide
ATU: allylthiourea
EC_50_: effective concentration 50
N: nitrogen
TN: total nitrogen
MBC: microbial biomass carbon
MBN: microbial biomass nitrogen
PNR: potential nitrification rate
WHC: water-holding capacity
BSA: bovine serum albumin
OTU: operational taxonomic unit
SIP: stable isotope probing
