# Supplementary material for: pH rather than nitrification and urease inhibitors determines the community of ammonia oxidizers in a vegetable soil
Source: AMB Express. 2017 Jun 21;7:129. doi: 10.1186/s13568-017-0426-x (PMC5479772; doi:10.1186/s13568-017-0426-x)
Supplement: Supplementary file 1 — Additional file 1: Table S1. Primers of AOA and AOB used for molecular analyses. Table S2. Pearson correlation between pH and the relative abundance of archaeal ammonia oxidizer TRFs. Table S3. Pearson correlation between pH and the relative abundance of bacterial ammonia oxidizer TRFs. Table S4. Genotype patterns based on the clone libraries of amoA genes. Figure S1. Log number of AOA and AOB amoA copies in four different treatments (control; urea; urea+nitrapyrin; urea+NBPT) at different pH levels. Error bars indicate standard errors of three replicates, different capital letters indicate the significant difference within different treatments at same pH level (P < 0.05). [file 13568_2017_426_MOESM1_ESM.docx]

**Supporting Information**

Applied Microbiology and Biotechnology Express

pH rather than nitrification and urease inhibitors determines the community of ammonia oxidizers in a vegetable soil

Ruijiao Xi^1,2,3^, Xi-En Long^1,2^, Sha Huang^1,3^, Huaiying Yao^1,2^ (🖂)

1. Key Laboratory of Urban Environment and Health, Institute of Urban Environment, Chinese Academy of Sciences, Xiamen 361021, People’s Republic of China
2. Ningbo Key Laboratory of Urban Environmental Processes and Pollution Control, Ningbo Urban Environment Observation and Research Station—NUEORS, Chinese Academy of Sciences, Ningbo 315800, People’s Republic of China
3. University of Chinese Academy of Sciences, Beijing 100049, People’s Republic of China

(🖂) Corresponding author. E-mail: [hyyao@iue.ac.cn](mailto:hyyao@iue.ac.cn) : Tel: +86-0592-6190791; Fax: +86-0592-6190791

Ruijiao Xi, E-mail: [rjxi@iue.ac.cn](mailto:rjxi@iue.ac.cn)

Xi-En Long, E-mail: xelong@iue.ac.cn

Sha Huang, E-mail: [shuang@iue.ac.cn](mailto:shuang@iue.ac.cn)

Table S1 Primers of AOA and AOB used for molecular analyses

| Target group | Primer | Sequence (5'-3') | | Length of amplicon (bp) | | Reference |
| --- | --- | --- | --- | --- | --- | --- |
| AOA | CrenamoA23f | | ATGGTCTGGCTWAGACG | | 635 | Nicol et al. (2008) |
|  | CrenamoA616r | | GCCATCCATCTGTATGTCCA | |  |  |
| AOB | amoA-1F | | GGGGTTTCTACTGGTGGT | | 491 | Rotthauwe et al. (1997) |
|  | amoA-2R | | CCCCTCKGSAAAGCCTTCTTC | |  |  |

Table S2 Pearson correlation between pH and the relative abundance of archaeal ammonia oxidizer TRFs

| Item | TRF256 | TRF217 | TRF205 | TRF166 | TRF123 | TRF79 | TRF54 |
| --- | --- | --- | --- | --- | --- | --- | --- |
| pH | 0.472 | 0.905** | 0.778** | -0.963** | -0.533* | -0.374 | -0.593* |

*Correlation is significant at the 0.05 level (two-tailed); **Correlation is significant at the 0.01 level (two-tailed)

Table S3 Pearson correlation between pH and the relative abundance of bacterial ammonia oxidizer TRFs

| Item | TRF256 | TRF238 | TRF157 | TRF56 |
| --- | --- | --- | --- | --- |
| pH | 0.510* | -0.271 | 0.721** | -0.825** |

*Correlation is significant at the 0.05 level (two-tailed); **Correlation is significant at the 0.01 level (two-tailed)

Table S4 Genotype patterns based on the clone libraries of *amoA* genes

| AOA T-RF | OTU | Representative sequences | Number of clones | Classification |
| --- | --- | --- | --- | --- |
| TRF 79 | OTU04 | AOA11 | 8 | *Nitrosophaeria* |
| TRF 79 | OTU06 | AOA10 | 1 | Cluster I |
| TRF 166 | OTU01 | AOA01 | 49 | Cluster II |
| TRF 205 | OTU05 | AOA18 | 3 | *Nitrosophaeria* |
| TRF 205 | OTU01 | AOA58 | 1 | Cluster II |
| TRF 217 | OTU02 | AOA09 | 24 | Cluster II |
| TRF 217 | OTU03 | AOA67 | 1 | *Nitrosophaeria* |
| TRF 256 | OTU03 | AOA02 | 13 | *Nitrosophaeria* |

| AOB T-RF | OTU | Representative sequences | Number of clones | Classification |
| --- | --- | --- | --- | --- |
| TRF 56 | OTU01 | AOB01 | 7 | Cluster I |
| TRF 56 | OTU02 | AOB29 | 5 | *β-proteobacteria* |
| TRF 56 | OTU13 | AOB07 | 5 | *β-proteobacteria* |
| TRF 56 | OTU14 | AOB11 | 5 | *β-proteobacteria* |
| TRF 56 | OTU15 | AOB21 | 5 | *β-proteobacteria* |
| TRF 56 | OTU16 | AOB94 | 1 | *β-proteobacteria* |
| TRF 56 | OTU03 | AOB73 | 1 | Cluster I |
| TRF 157 | OTU07 | AOB12 | 3 | *β-proteobacteria* |
| TRF 157 | OTU02 | AOB95 | 1 | *β-proteobacteria* |
| TRF 157 | OTU12 | AOB34 | 1 | *β-proteobacteria* |
| TRF 157 | OTU15 | AOB70 | 1 | *β-proteobacteria* |
| TRF 157 | OTU04 | AOB03 | 5 | Cluster I |
| TRF 157 | OTU01 | AOB60 | 1 | Cluster I |
| TRF 157 | OTU05 | AOB02 | 44 | Cluster II |
| TRF 157 | OTU08 | AOB22 | 2 | Cluster II |
| TRF 157 | OTU09 | AOB72 | 1 | Cluster II |
| TRF 157 | OTU10 | AOB47 | 1 | Cluster II |
| TRF 157 | OTU06 | AOB17 | 1 | Cluster III |
| TRF 157 | OTU11 | AOB76 | 1 | Cluster III |
| TRF 235 | OTU01 | AOB92 | 1 | Cluster I |
| TRF 235 | OTU05 | AOB74 | 1 | Cluster II |
| TRF 256 | OTU06 | AOB100 | 1 | Cluster III |
| TRF 235 | OTU08 | AOB04 | 7 | Cluster II |


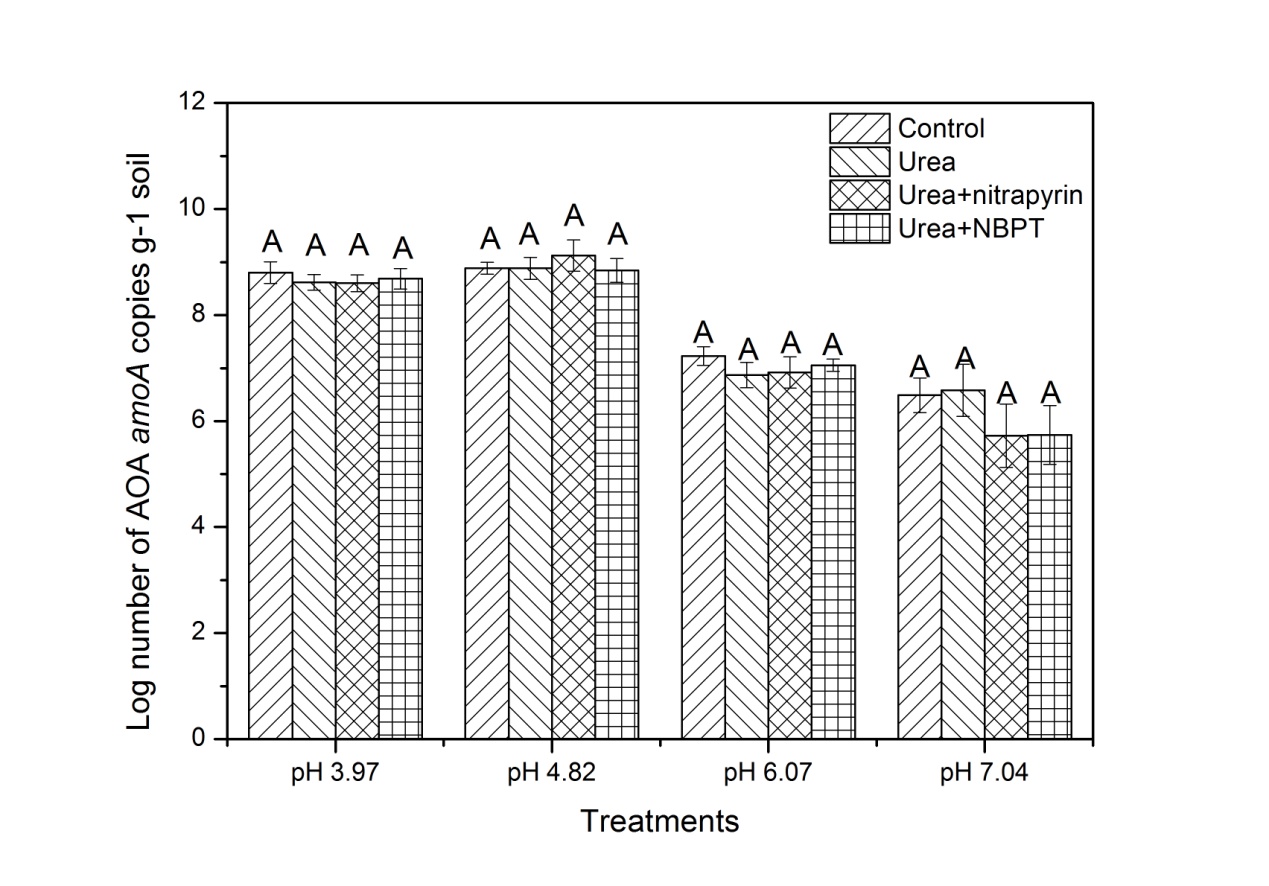


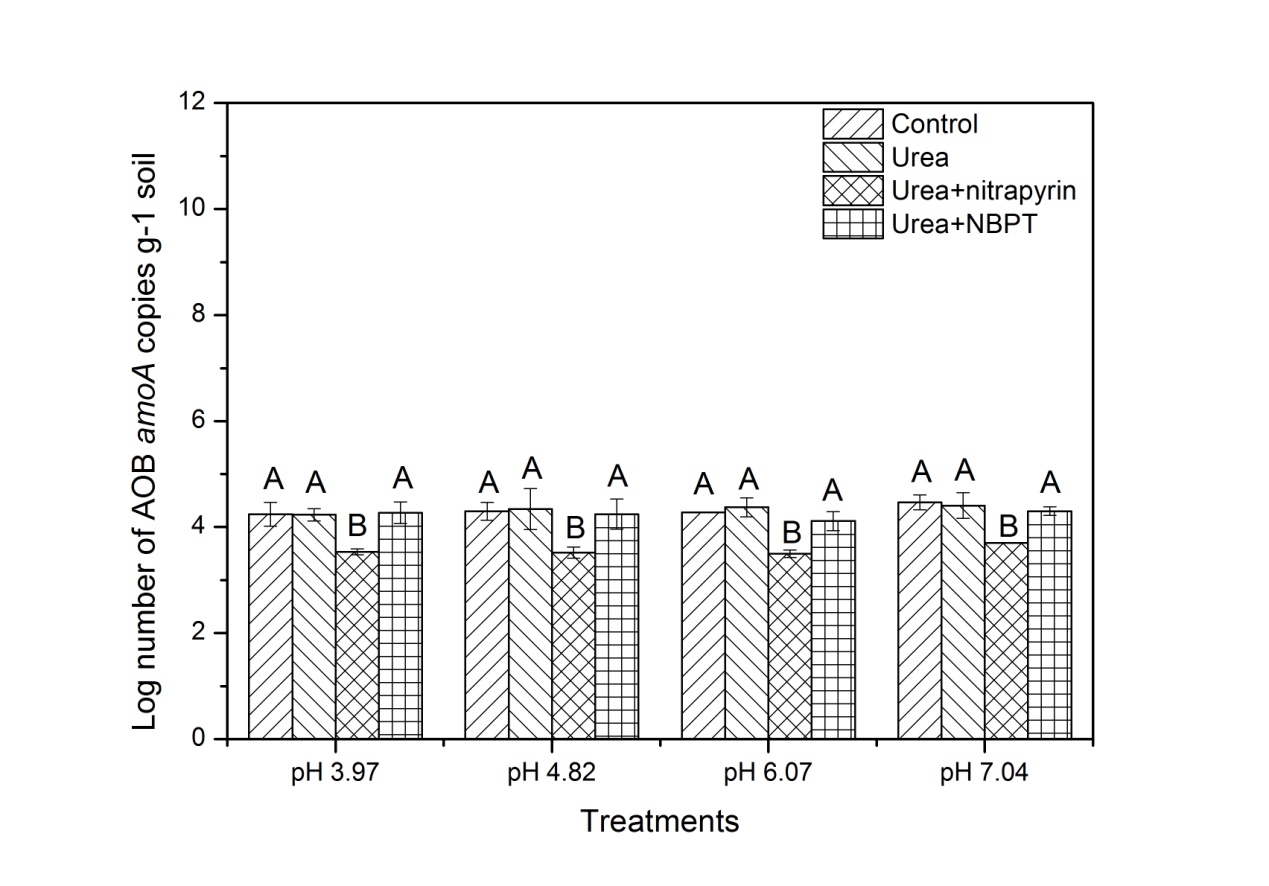
 Fig. S1 Log number of AOA and AOB *amoA* copies in four different treatments (control; urea; urea+nitrapyrin; urea+NBPT) at different pH levels. Error bars indicate standard errors of three replicates, different capital letters indicate the significant difference within different treatments at same pH level (*P*<0.05)

**References**

Nicol GW, Leininger S, Schleper C, Prosser JI (2008) The influence of soil pH on the diversity, abundance and transcriptional activity of ammonia oxidizing archaea and bacteria. Environ Microbiol 10:2966–2978

Rotthauwe JH, Witzel KP, Liesack W (1997) The ammonia monooxygenase structural gene *amoA* as a functional marker: molecular fine-scale analysis of natural ammonia-oxidizing populations. Appl Environ Microbiol 63:4704-4712
